# Increased Human Interleukin-32 Expression Is Related to Disease Activity of Graves' Disease

**DOI:** 10.3389/fendo.2019.00613

**Published:** 2019-09-26

**Authors:** Qiuming Yao, Bin Wang, Xi Jia, Qian Li, Wei Yao, Jin-an Zhang

**Affiliations:** ^1^Department of Endocrinology, Jinshan Hospital of Fudan University, Shanghai, China; ^2^Department of Endocrinology & Rheumatology, Shanghai University of Medicine & Health Sciences Affiliated Zhoupu Hospital, Shanghai, China

**Keywords:** interleukin-32, CD4^+^IL-32α^+^T cells, Graves' disease, cytokine, thyroid

## Abstract

Recently, abnormal expression of interleukin-32 (IL-32) has been involved in various inflammatory or autoimmune diseases, but the level of IL-32 expression in Graves' disease (GD) is still unknown. This study is aimed to explore the human IL-32 expression in GD and the association of IL-32 expression with the disease activity of GD. A total of 125 GD patients and 97 normal controls (NC) were recruited in this study. We examined IL-32 mRNA level in peripheral blood mononuclear cells (PBMCs) of 43 GD patients and 41 controls using real-time polymerase chain reaction (RT-PCR). Serum IL-32 level of 40 GD patients and 34 controls was measured by enzyme linked immunosorbent assay (ELISA). In another cohort including 42 GD patients and 22 controls, we detected the percentages of IL-32α^+^ cells, CD4^+^IL-32α^+^T cells, and CD4^−^IL-32α^+^ cells in PBMCs by flow cytometry. In GD patients, IL-32 mRNA expression was dramatically higher than that in controls (*P* < 0.001) and positively associated with FT3 (*P* = 0.036, *r* = 0.321). Subgroup analysis revealed that IL-32 mRNA level was elevated in both newly onset GD and refractory GD group (*P* < 0.01, *P* < 0.001, respectively) compared with controls. Furthermore, in refractory GD group, the IL-32 mRNA expression also positively correlated with FT3 (*P* = 0.019, *r* = 0.560). In addition, serum IL-32 level was notably higher in GD patients than that of controls (*P* < 0.01). Subgroup analysis also indicated that serum IL-32 level in both newly onset GD and refractory GD group was higher in comparison with controls (*P* = 0.015, *P* = 0.023, respectively) and serum IL-32 level in refractory GD patients positively correlated with TRAb (*P* = 0.043, *r* = 0.481). The percentages of IL-32α^+^ cells, CD4^+^IL-32α^+^ T cells, and CD4^−^IL-32α^+^ cells were all significantly enhanced in GD patients compared with controls (*P* = 0.005, *P* = 0.017, *P* = 0.016, respectively). IL-32 and IL-32α^+^ cells may be associated with the pathogenesis of GD. IL-32 may become a promising target for the treatment of GD.

## Introduction

Graves' disease (GD) is a common organ-specific autoimmune disease characterized by hyperthyroidism and positive serum thyrotrophin receptor antibody (TRAb). The incidence peak of GD is between 30 and 50 years of age, but people can suffer from GD at any age (1). The ratio of male to female GD is about 1–6 (1). The clinical manifestations of GD are mainly thyrotoxicosis and diffuse goiter. Symptoms of some patients are accompanied by ophthalmopathy and dermopathy. In addition, after treatment with antithyroid drugs, the recurrence rate of GD is as high as 30–60% (2, 3). Furthermore, GD can be associated with other systemic autoimmune diseases. It has been reported that the most common autoimmune disease associated with GD patients is vitiligo, followed by chronic autoimmune gastritis and rheumatoid arthritis (4). Obviously, the life quality of patients with GD will decline significantly. Many studies have shown that a variety of cytokines are related to GD's pathogenesis and can influence its severity or prognosis (5–9), and peroxisome proliferator activated receptor (PPAR)-γ and -α agonists can modulate CXCR3 chemokines that are implicated in the pathogenesis of Graves' disease (10, 11). Despite this, the pathogenesis of GD is still elusive.

Interleukin 32 (IL-32), originally identified in human natural killer (NK) cells and T cells stimulated with IL-2 or mitogens, was initially called NK cell transcript 4 (NK4) (12). It is expressed in endothelial cells, epithelial cells, and immune cells (NK cells, T cells, and dendritic cells) (13, 14). The gene encoding IL-32 is located on chromosome 16p13.3 and has multiple functions. It is associated with the death of T cells (15), and can induce the differentiation of monocytes (16), and promote production of TNF-α and IL-1β (17). Recently, abnormal expression of IL-32 has been linked to various inflammatory or autoimmune diseases, including rheumatoid arthritis (RA) (18–20), inflammatory bowel disease (IBD) (21), systemic lupus erythematosus (SLE) (22), allergic rhinitis (23), Behcet's disease (24), psoriasis and psoriatic arthritis (25). But its role in GD remains largely unknown.

In this study, we firstly explored the association of IL-32 with Graves' disease. We investigated the IL-32 expression in GD and analyzed the association between expression level of IL-32 and thyroid function.

## Materials and Methods

### Subjects

We enrolled a total of 125 GD patients, including 70 newly onset and 55 refractory GD patients, and 97 healthy controls in this study. All patients were collected from the Outpatient of the Department of Endocrinology, Zhoupu Hospital, Shanghai, China. All healthy controls were obtained from the health examination center of the same hospital. GD was diagnosed based on thyrotoxicosis, elevated free triiodothyronine (FT3), increased free thyroxine (FT4), decreased thyroid-stimulating hormone (TSH), and positive serum antibodies to TRAb. GD patients with other autoimmune diseases or Graves' ophthalmopathy were excluded. Newly onset GD patients were those who had just been diagnosed with GD but had not yet received any drug treatment. GD patients who have received anti-thyroid medication treatment regularly for at least 2 years and still positive for TRAb were defined as refractory patients (26). All healthy controls had no other autoimmune, infectious, or thyroid diseases. Among them, 43 GD patients, including 26 newly onset GD patients (40.5 ± 13.0 years, 9 males and 17 females) and 17 refractory GD patients (35.2 ± 11.5 years, 3 males and 14 females), along with 41 normal subjects (36.1 ± 10.7 years, 15 males and 26 females) were recruited for PCR analysis. Another 40 GD patients, including 22 newly onset GD patients (38.2 ± 14.0 years, 7 males and 15 females) and 18 refractory GD patients (38.6 ± 10.6 years, 4 males and 14 females), along with 34 normal subjects (35.0 ± 11.8 years, 14 males and 20 females) were recruited for ELISA. Another 42 GD patients, including 22 newly onset GD patients (39.4 ± 13.5 years, 8 males and 14 females) and 20 refractory GD patients (39.9 ± 14.7 years, 7 males and 13 females), along with 22 normal subjects (37.8 ± 11.0 years, 9 males and 13 females) were enrolled for flow cytometry. We summarized the clinical information of all subjects in Table 1. This study was permitted by the Ethics Review Board of Zhoupu Hospital. All subjects signed informed consent.

**Table 1:** Clinical data of all subjects.

**Research method**	**Group**	**N (M/F)**	**Age (years)**	**FT3 (pmol/L)**	**FT4 (pmol/L)**	**TSH (mIU/L)**	**TRAb (IU/L)**
qRT-PCR	Newly onset GD	26 (9/17)	40.5 ± 13.0	18.2 ± 11.4	40.0 ± 24.1	<0.01	10.7 ± 10.5
	Refractory GD	17 (3/14)	35.2 ± 11.5	16.1 ± 14.7	28.9 ± 16.7	<0.01	14.2 ± 11.9
	Controls	41 (15/26)	36.1 ± 10.7	–	–	–	–
ELISA	Newly onset GD	22 (7/15)	38.2 ± 14.0	26.3 ± 11.8	72.7 ± 27.0	<0.01	14.8 ± 13.1
	Refractory GD	18 (4/14)	38.6 ± 10.6	16.5 ± 10.3	44.6 ± 26.7	<0.01	15.2 ± 14.0
	Controls	34 (14/20)	35.0 ± 11.8	–	–	–	–
Flow cytometry	Newly onset GD	22 (8/14)	39.4 ± 13.5	17.3 ± 13.4	36.8 ± 22.1	<0.01	12.0 ± 9.4
	Refractory GD	20 (7/13)	39.9 ± 14.7	12.8 ± 12.2	24.8 ± 11.7	<0.01	10.2 ± 8.2
	Controls	22 (9/13)	37.8 ± 11.0	–	–	–	–

*Data were expressed as M ± SD; M, Male; F, Female; FT3, free Triiodothyronine; FT4, free Thyroxine; TSH, thyroid-stimulating hormone; TRAb, thyrotropin receptor antibody; qRT-PCR, quantitative real-time polymerase chain reaction; ELISA, enzyme linked immunosorbent assay*.

### Isolation of Peripheral Blood Mononuclear Cells (PBMCs)

PBMCs isolation was performed using lymphocyte separation medium according to the manufacturer's instruction. PBMCs from 2 mL EDTA anticoagulated peripheral venous blood were used for RNA extraction and PBMCs isolated from 5 ml blood preserved in heparin sodium were used for flow cytometry.

### Quantitative Real-Time Polymerase Chain Reaction (qRT-PCR)

We extracted the total RNA from PBMCs using Trizol reagent (TakaRa). After that, we used Primescript RT reagent kit (TaKaRa) to convert 1 μg total RNA into cDNA, which was then stored at −20°C. The qRT-PCR was performed using IL-32α primer pair TGGCGGCTTATTATGAGGAGC and CTCGGCACCGTAATCCATCTC with SYBR Premix Ex TaqTM II (TaKaRa) in ABI PRISM 7300. The PCR condition was 95°C for 30 s, 5 s at 95°C for 40 cycles, followed by 63°C for 31 s. β-actin expression was used to normalize the gene expression of IL-32.

### Plasma Separation and Plasma IL-32 Assay

The above EDTA anticoagulant blood was centrifuged for 5 min at 4,500 rpm. We collected the supernatant and centrifuged it again at 13,000 rpm for 2 min. The plasma was then obtained. The serum IL-32 level was measured using a commercial ELISA kit (R&D Systems, USA).

### Flow Cytometric Analysis

We incubated 1.0–2.0 × 10^6^/ml PBMCs at 37°C for 5 h with leukocyte activation cocktail (Cat# 550583, BD Biosciences Pharmingen). After being washed in staining buffer (Cat. No. 554657) (BD Biosciences Pharmingen), cells were stained in the dark with fluorescein isothiocyanate (FITC)-conjugated anti-CD4 (Cat# 555346, BD Biosciences Pharmingen) for 20 min at 4°C. After being washed again with staining buffer, cells were fixed and permeabilized in the dark using a cytofix/cytoperm kit (Cat# 554714, BD Biosciences Pharmingen) for 20 min at 4°C. After being washed with wash buffer, cells were incubated in the dark with phycoerythrin (APC)-conjugated anti-IL-32α (Cat# IC30402A, R&D Systems, USA) or isotype control Ab at 4°C for 30 min. Finally, after being washed again with wash buffer, cells were immediately analyzed on a flow cytometer (Beckman coulter).

### Statistical Analysis

We analyzed all data in this study using the SPSS 17.0. All continuous data were displayed as mean ± standard deviation (M ± SD). We used Non-parametric Mann–Whitney *U*-test to analyze the statistical difference of non-normally distributed data between two groups. One-way analysis of variance was used to compare differences between the three groups. We performed correlation analysis by Spearman rank correlation test. A *p*-value < 0.05 in two tailed analysis was considered to be statistically significant.

## Results

### IL-32 mRNA Level in PBMCs

**Figure 1:**
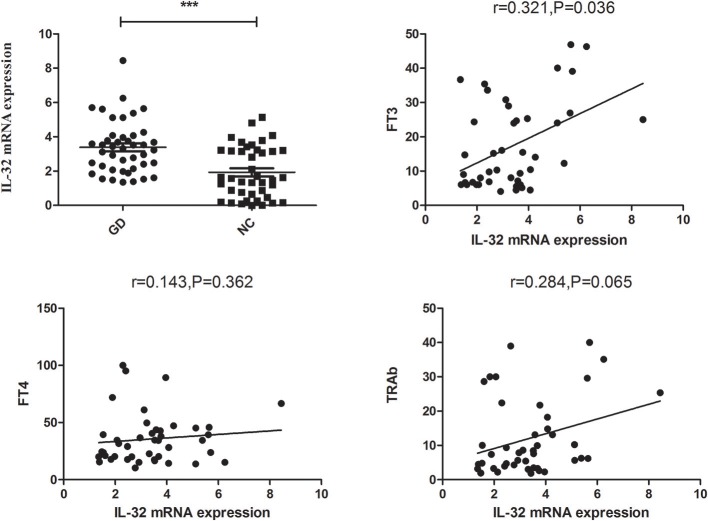
The expression of IL-32 mRNA in GD patients. IL-32 mRNA expression in GD patients was significantly higher than that in controls; IL-32 mRNA expression in GD patients was positively correlated FT3. NC, normal controls. ***P < 0.001.

Figure 1 showed that IL-32 mRNA expression was prominently increased in GD patients compared with that of healthy controls (*P* < 0.001). Correlation analysis indicated that IL-32 mRNA level was positively associated with FT3 (*P* = 0.036, *r* = 0.321). No correlation was found between IL-32 mRNA and FT4 as well as TRAb (*P* > 0.05).

**Figure 2:**
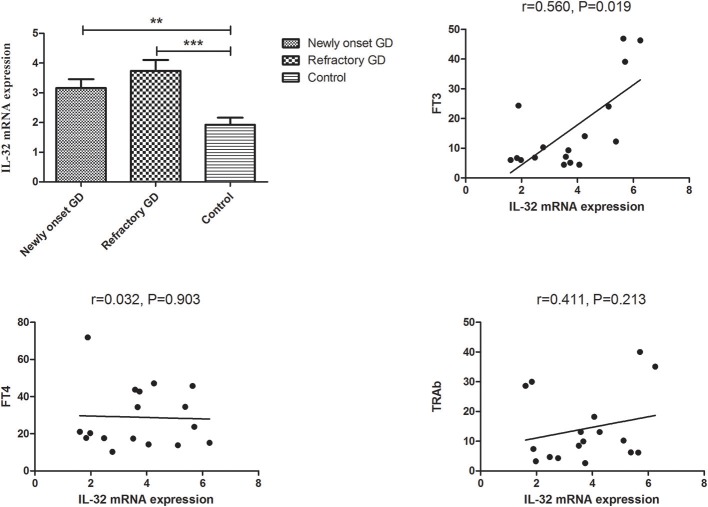
Subgroup analysis of the expression of IL-32 mRNA in newly onset GD patients and refractory GD patients. IL-32 mRNA expression in both newly onset GD patients and refractory GD patients was significantly higher than that in controls; IL-32 mRNA expression in refractory GD patients was positively correlated with FT3. **P < 0.01, ***P < 0.001.

As illustrated in Figure 2, subgroup analysis also revealed that the IL-32 mRNA expression in both newly onset GD and refractory GD group was higher in comparison with controls (*P* < 0.01, *P* < 0.001, respectively). Furthermore, correlation analysis also showed that in refractory GD group, IL-32 mRNA expression also positively correlated with FT3 (*P* = 0.019, *r* = 0.560).

### Serum IL-32α Levels

**Figure 3:**
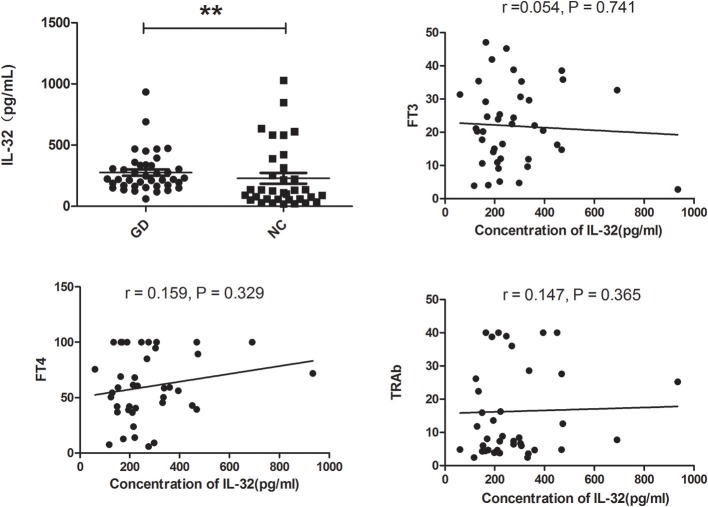
Serum IL-32 levels of GD patients and healthy controls. Serum IL-32 level of GD patients was significantly elevated compared with controls. No correlation was found between IL-32 concentration and FT3, FT4, and TRAb (all *P* > 0.05). **P < 0.01.

Figure 3 showed that serum IL-32 concentration was higher in GD group compared with controls (*P* < 0.01). In addition, no correlation was found between serum IL-32 level and TRAb, FT3 as well as FT4 (all *P* > 0.05, Figure 3).

**Figure 4:**
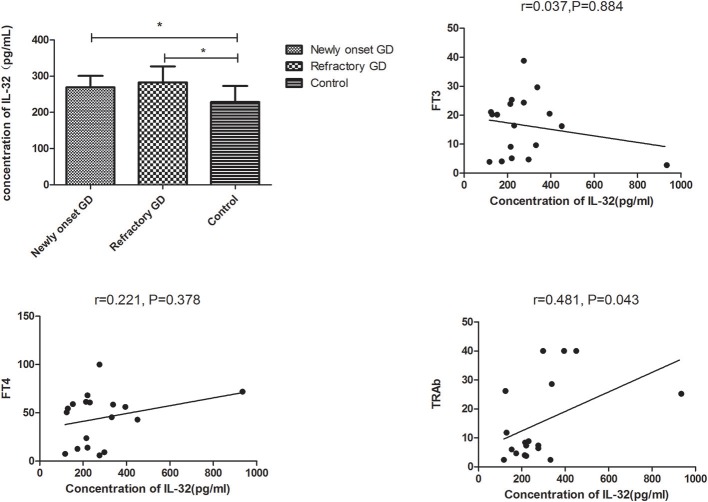
Subgroup analysis of the concentration of IL-32 in the plasma of newly onset GD patients and refractory GD patients. IL-32 concentration in both newly onset GD patients and refractory GD patients was higher than that in controls; IL-32 mRNA expression in refractory GD patients was positively correlated with TRAb. In refractory GD patients, no correlation was found between IL-32 concentration and FT3 and FT4 (all ^*^P > 0.05).

Subgroup analysis indicated that IL-32 concentration in both newly onset GD and refractory GD group was also higher than that of controls (*P* = 0.015, *P* = 0.023, respectively, Figure 4). Furthermore, serum IL-32 concentration in refractory GD patients was positively associated with TRAb (*P* = 0.043, *r* = 0.481, Figure 4). But no association was determined between serum IL-32 level and FT3, FT4 as well as TRAb in newly onset GD group (all *P* > 0.05, data not shown).

### Frequency of CD4^+^IL-32α^+^ T Cells in PBMCs

**Figure 5:**
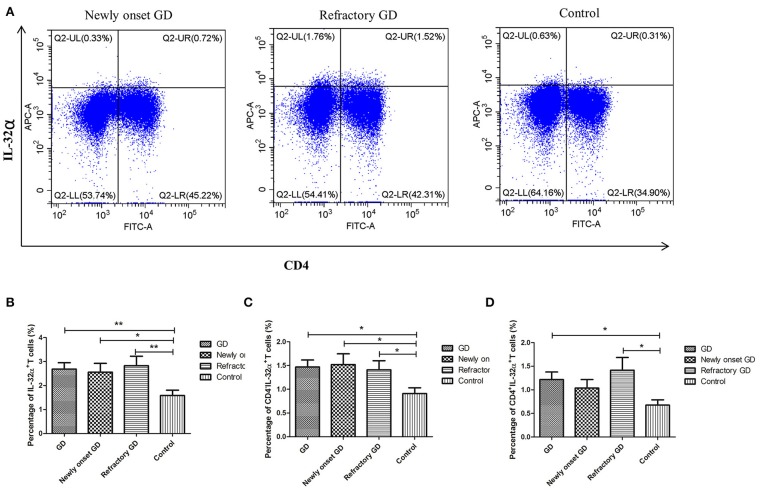
Flow cytometric analysis detected IL-32α^+^ cells. **(A)** Representative flow cytometric data showing the expression of IL-32α^+^ cells in newly onset GD patients, refractory GD patients and controls. **(B)** The frequency of IL-32α^+^ cells in GD group, newly onset GDs group, refractory GD group and controls. **(C)** The frequency of CD4^−^IL-32α^+^ cells in GD group, newly onset GDs group, refractory GD group and controls. **(D)** The frequency of CD4^+^IL-32α^+^ T cells in GD group, newly onset GDs group, refractory GD group, and controls *P < 0.05, **P < 0.01.

As shown in Figure 5, the percentage of IL-32α^+^ cells, CD4^+^IL-32α^+^ T cells, and CD4^−^IL-32α^+^ cells were all elevated in GD group compared with controls (*P* = 0.005, *P* = 0.017, *P* = 0.016 respectively). Subgroup analysis displayed that the percentage of IL-32α^+^ cells and CD4^−^IL-32α^+^ T cells, but not CD4^+^IL-32α^+^ T cells of newly onset GD group, were increased compared with controls (*P* = 0.03, *P* = 0.037, *P* = 0.057, respectively). In refractory GD group, the percentage of IL-32α^+^ cells, CD4^+^IL-32α^+^ T cells, and CD4^−^IL-32α^+^ cells were all significantly elevated compared with controls (*P* = 0.009, *P* = 0.028, *P* = 0.040, respectively). Correlation analysis revealed that the percentage of IL-32α^+^ cells, CD4^+^IL-32α^+^ T cells, and CD4^−^IL-32α^+^ cells in GD group (including newly onset GD and refractory GD group) were not associated with FT3, FT4, and TRAb (all *P* > 0.05, data not shown).

## Discussion

In this study, we firstly revealed that IL-32 mRNA and IL-32α^+^ cells in PBMCs of GD patients were dramatically increased compared with that of healthy controls. Also, our study was the first to explore the serum IL-32 level of GD group and found that GD patients showed higher serum IL-32 concentration compared with that of controls. In addition, we found that IL-32 level of serum was positively associated with TRAb in refractory GD patients.

IL-32 has at least four different spliced isoforms and IL-32α is the most abundant and shortest among them (27). IL-32 has been found to be associated with various autoimmune diseases. In myasthenia gravis (MG) patients, serum IL-32α level was significantly higher compared with healthy controls and tended to reduce with clinical improvement (27). A recent study also reported that IL-32 mRNA level was increased in PBMCs of chronic psoriatic patients and its four isoforms, including α, β, γ, and δ, were all overexpressed compared with the controls (28). The IL-32 mRNA expression in PBMCs of active RA groups was positively correlated with TNF-α mRNA expression and the serum IL-32α level in active RA group was significantly related to TNF-α and other Key clinical indicators (29). Our results were in accordance with the above findings. All these findings further indicate that IL-32 might play a crucial role in the pathogenesis of autoimmune diseases. However, Wang et al. revealed that plasma IL-32 level was lower in SLE group than in healthy controls (22). The authors speculated that this outcome may be linked to medication treatment because most SLE patients enrolled in their study had received at least 6 months of medication therapy. Therefore, research design and sample selection have significant impacts on the research results.

Our study firstly revealed that the IL-32 mRNA expression in PBMCs was notably higher in GD group than in normal controls. Correlation analysis showed that IL-32 mRNA expression in GD group was positively associated with FT3, suggesting that IL-32 was related to the occurrence of GD and function of thyroid. Subgroup analysis also displayed that the mRNA expression of IL-32 was higher in both newly onset GD and refractory GD patients than in controls. IL-32 mRNA expression was also significantly positively correlated with FT3 in the refractory GD group. The results of ELISA were consistent with those of PCR. We found that serum IL-32 level was significantly increased in GD group. Subgroup analysis also indicated that IL-32 concentration in both newly onset GD and refractory GD patients was elevated in comparison with controls. Furthermore, serum IL-32 concentration in refractory GD group was positively associated with TRAb, indicating that IL-32 may be related to the severity of GD. To further study the IL-32 expression in GD, we performed flow cytometry to analyze the percentage of IL-32α^+^ cells in PBMCs. Our results indicated that the percentage of IL-32α^+^ cells was higher in GD group compared with controls. Further analysis demonstrated that both CD4^−^IL32α^+^ cells and CD4^+^IL-32α^+^T cells in GD patients were increased compared with controls, indicating that CD4^+^ T cells are one of the cells which can produce IL-32α in GD, and other PBMCs such as monocytes and B cells may also secrete IL-32.

Our study firstly showed high IL-32 expression in GD patients and indicated that IL-32 is related to the pathogenesis of GD. But the specific mechanism of IL-32 in GD is still unclear. Further studies are needed to determine the important role of IL-32 in GD. For example, one can knock out IL-32 gene in mice to explore the effect of IL-32 knockout on the success rate of constructing GD model, as well as the change in the percentage of T cells, etc.

In conclusion, our preliminary findings suggested that abnormal expression of IL-32 may be associated with the occurrence and development of GD. In the future, targeting IL-32α may be a promising treatment method for GD. Further studies are also needed to expand the current observations, especially when further investigating the mechanism of IL-32α in GD.

## Data Availability

All datasets generated for this study are included in the manuscript/supplementary files.

## Ethics Statement

The studies involving human participants were reviewed and approved by Ethics Review Board of Zhoupu Hospital. The patients/participants provided their written informed consent to participate in this study.

## Author Contributions

QY collected data, performed statistical analyses, and wrote the final version of the manuscript. BW, XJ, and QL participated in subjects collection. WY and JZ designed the study and revised the manuscript. All authors approved the final version of the manuscript.

### Conflict of Interest Statement

The authors declare that the research was conducted in the absence of any commercial or financial relationships that could be construed as a potential conflict of interest.
